# Identifying rare variants for quantitative traits in extreme samples of population via Kullback-Leibler distance

**DOI:** 10.1186/s12863-020-00951-2

**Published:** 2020-11-24

**Authors:** Yang Xiang, Xinrong Xiang, Yumei Li

**Affiliations:** 1 School of Mathematics and Computational Science, Huaihua University, Huaihua, Hunan 418008 People’s Republic of China; 2 Key Laboratory of Research and Utilization of Ethnomedicinal Plant Resources of Hunan Province, Huaihua University, Huaihua, 418008 China; 3 Key Laboratory of Hunan Higher Education for Western Hunan Medicinal Plant and Ethnobotany, Huaihua University, Huaihua, 418008 China; 4 School of Mathematics and Statistics, Hunan Normal University, Changsha, Hunan 410081 People’s Republic of China

**Keywords:** Quantitative trait, Rare variant, Association analysis, Fine mapping, Extreme phenotype

## Abstract

**Background:**

The rapid development of sequencing technology and simultaneously the availability of large quantities of sequence data has facilitated the identification of rare variant associated with quantitative traits. However, existing statistical methods depend on certain assumptions and thus lacking uniform power. The present study focuses on mapping rare variant associated with quantitative traits.

**Results:**

In the present study, we proposed a two-stage strategy to identify rare variant of quantitative traits using phenotype extreme selection design and Kullback-Leibler distance, where the first stage was association analysis and the second stage was fine mapping. We presented a statistic and a linkage disequilibrium measure for the first stage and the second stage, respectively. Theory analysis and simulation study showed that (1) the power of the proposed statistic for association analysis increased with the stringency of the sample selection and was affected slightly by non-causal variants and opposite effect variants, (2) the statistic here achieved higher power than three commonly used methods, and (3) the linkage disequilibrium measure for fine mapping was independent of the frequencies of non-causal variants and simply dependent on the frequencies of causal variants.

**Conclusions:**

We conclude that the two-stage strategy here can be used effectively to mapping rare variant associated with quantitative traits.

## Background

Thanks to the rapid development of sequencing technology and the lowering of sequencing costs in the last decade, the availability of large quantities of sequence data provides an unprecedented opportunity for researchers to investigate the role of rare variants in complex traits [1–4]. But due to the low minor allele frequency (MAF < 5%) and thus resulting in weak linkage disequilibrium (LD) with nearby markers, detecting rare variant (RV) association with complex traits faces great challenges [5–8]. One challenge is that detection of rare causal variants with traditional designs usually requires a large sample, which will be the high cost [3, 6]. Thus cost-effective design should be considered to reduce sample size. Another challenge is that the statistical power with test statistics of single-marker tests is generally low in genetic association studies of rare variants with more moderate or weak genetic effects [8–10]. To date many statistical methods have been developed for rare variant association analysis, including burden tests [11–13], variance-component tests [14, 15], series of sequence kernel association tests [10, 16, 17]. Any of these methods has relative perfect performance in special scenario, but none of them can overwhelm others in all scenarios [8, 9], especially for quantitative traits.

In fact, rare variant association analysis in the past several years mainly focused on the qualitative trait. Only a few statistical methods have been developed for the quantitative trait [13, 18–21]. One approach for rare variant association analysis of quantitative traits is the linear regression model. However, most regression-based methods rely on the normality assumption of the phenotype [8, 21]. Another commonly used approach adopts phenotype extreme selection design where one can transform the quantitative trait association study into case-control association study of qualitative traits by treating the upper extreme as cases and the lower extreme as controls in a strategy using extreme phenotype [22–25]. Extreme phenotypes of a quantitative trait are generally considered to be more informative. Moreover, a smaller sample size for extreme-phenotype sampling than that for random sampling is needed to achieve similar power [23, 24].

In this report, we use phenotype extreme selection design and Kullback-Leibler distance (KL-distance) [26] to propose a simple statistic method to identify rare variants for quantitative traits. Two stages strategies are adopted in our analysis where association analysis and fine mapping will be done in the first stage and the second stage, respectively. This method will compare the frequency distributions of rare variant in two extreme phenotypes based on KL-distance. Our method has three features: (1) it has increasing power with the stringency of the sample selection, (2) it is affected slightly by non-causal variants and the opposite effect variants.in the first stage for association analysis, and (3) it is not depend on the frequencies of non-causal variants and just dependent on the frequencies of causal variants in the second stage for fine mapping. Through simulation studies, we investigate the performance of the proposed method and compare it with three commonly used methods of the burden test [12], the sequence kernel association test (SKAT) [17], and the optimal test that combines SKAT and the burden test (SKAT-O) [10].

## Results

### Type I error rate and power for association analysis

Table 1 exhibits the estimated type I error rates of the statistic T_KL_ for the extreme sample with sample-selection threshold value of 20, 10, and 5% and with sample size of 1000 and 1500. It can be seen that, under various genetic parameters, type I error rates of T_KL_ are not appreciably different from the nominal alpha levels, which indicates the validity of the statistic T_KL_.

**Table 1:** Estimated type I error rates of the statistic T_KL_

Threshold values	Estimated Type I error rate			
	2 *N* = 1000		2 *N* = 1500	
	*α* = .05	*α* = .01	*α* = .05	*α* = .01
20%	0.048	0.012	0.048	0.011
10%	0.049	0.014	0.051	0.013
5%	0.053	0.009	0.052	0.013

**Fig. 1:**
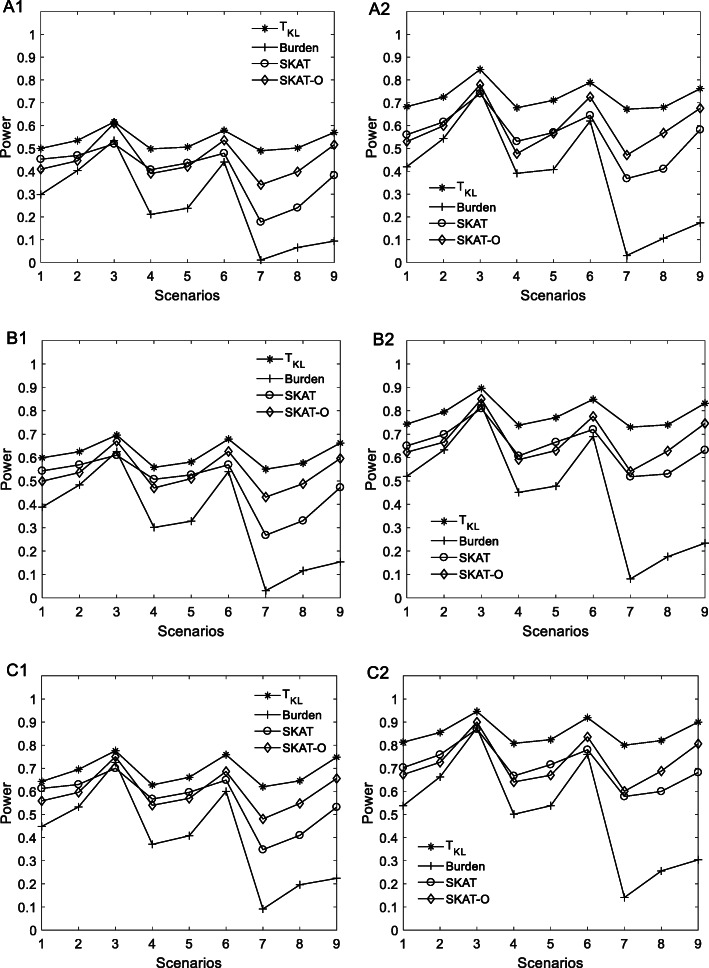
Empirical power of four statistics from the extreme samples with 20% threshold value **a**, 10% threshold value **b**, and 5% threshold value **c** when the sample sizes are 1000 (**a1, b1, c1**) and 1500 (**a2, b2, c2**) at a 0.05 significance level

Figure 1 shows the results of power for 9 scenarios when sample sizes are 1000 and 1500. It is found that the power of the statistic T_KL_ with the sample size of 1500 is nearly 0.20 larger than that with the sample size of 1000, indicating that the power of the statistic T_KL_ significantly increase with the increasing of the sample size. It can be seen that, under the same sample size, the powers of the statistic T_KL_ with the low 5% samples and the up 5% samples are highest and the powers with the low 20% samples and the up 20% samples are lowest, which indicates that the powers of the statistic T_KL_ increase with the stringency of the sample selection. It is observed from scenarios {1, 2, 3} that, when rare variant effects are in the same direction, the powers of the statistic T_KL_ increase with the increasing of the number of causal variants. The same above conclusions are observed when 80% causal variants have positive effect and 20% causal variants have negative effect (scenarios {4, 5, 6}) and when there is the same number of causal variants with positive effects and negative effects (scenarios {7, 8, 9}). By comparing the powers under scenarios {1, 4, 7} with 10 causal variants, the powers under scenarios {2, 5, 8} with 20 causal variants, and the powers under scenarios {3, 6, 9} with 50 causal variants, we found that, when the number of causal variants with negative effect increases, the power of the statistic T_KL_ decreases slightly. From Fig. 1, we can observe that, among four statistics of the T_KL_, the burden test, the SKAT, and the SKAT-O, the power of T_KL_ is higher than that of other three statistics. The burden test, the SKAT, and the SKAT-O are severely affected by the number of non-causal variants and the opposite effect variants, especially when there are the same number of opposite effect variants. Although non-causal variants and the opposite effect variants affect the power of the T_KL_, the impact is slight. For example, when the sample size is 1500 and the number of causal variants is 50 for 10% sample-selection threshold value (B2), as the number of variants with negative effect increases from zero to 25, the powers of the burden test, the SKAT, and the SKAT-O decrease from ~ 0.80, ~ 0.79, and ~ 0.84 to ~ 0.23, ~ 0.63, and ~ 0.74, respectively, with the decline rate of 71.2, 20.2, and 12.0%. Nevertheless, when the number of variants with negative effect is 25, the T_KL_ still achieves ~ 0.83 power, with the decline rate of just 7% comparing to ~ 0.90 when the number of variants with negative effect is zero.

### Power for fine mapping

In fine mapping study, the QTL can be located by the maximum value of the measure *l*_*KL*_. So we sample 10 times from each of 100 simulation populations where each sample includes 750 individuals with the up-extreme phenotype of Y > *U* and 750 individuals with the lower-extreme phenotype with Y < *L*. For each sample, we calculate the value of the measure *l*_*KL*_ for each variant. In order to guard against noisy distributions of the measure *l*_*KL*_, we adopt the 5-point moving-average method to determine the maximum value. We count the number (here, we denote it B) of the maximum values that locate at variant 10 or variant 11. Then the probability that the maximum values of *l*_*KL*_ locate at variant 10 or variant 11 is B/1000. We refer this value as the power of *l*_*KL*_, which measure the likelihood of fine mapping the QTL. Table 2 shows the results of the power for *l*_*KL*_. It can be seen that the power of *l*_*KL*_ for fine mapping under dominant model is highest and the power of *l*_*KL*_ for fine mapping under recessive model is lowest. The power of *l*_*KL*_ increases with increasing of the heritability h^2^ of the causal variant and the stringency of the sample selection. For example, power of *l*_*KL*_ under dominant model with the heritability h^2^ of 0.01 is 0.52, 0.62, and 0.67 at 20, 10, and 5% sample-selection threshold value, respectively; power of *l*_*KL*_ under dominant at 5% sample-selection threshold value increase from 0.67 to 0.83 with the heritability h^2^ of the causal variant increasing from 0.01 to 0.10. We also investigate the effect of different sample sizes (e.g., 2n =1000, 1500, and 2000). As expected, power of *l*_*KL*_ increases with the increasing sample size (data not shown). In order to assess the performance of *l*_*KL*_, we compare it with two LD measures *l* [27] and *p*_*excess*_ [28] with case-control design using extreme samples. Table 2 also lists the powers for *l* and *p*_*excess*_. We found that the powers of *l*_*KL*_ and *l* are nearly the same and higher than those of *p*_*excess*_.

**Table 2:** The power of the QTL fine mapping for three LD measures by use of five-point moving average

Sample-selection threshold values	Power of the QTL fine mapping								
	Recessive model			Additive model			Dominant model		
	*h*^2^ = 0.01	*h*^2^ = 0.05	*h*^2^ = 0.10	*h*^2^ = 0.01	*h*^2^ = 0.05	*h*^2^ = 0.10	*h*^2^ = 0.01	*h*^2^ = 0.05	*h*^2^ = 0.10
20%									
*l*_*KL*_	0.39	0.50	0.58	0.44	0.53	0.62	0.52	0.59	0.70
*l*	0.40	0.49	0.59	0.45	0.53	0.62	0.51	0.60	0.70
*p*_*excess*_	0.29	0.36	0.47	0.36	0.47	0.58	0.41	0.52	0.61
10%									
*l*_*KL*_	0.49	0.59	0.66	0.52	0.64	0.70	0.62	0.69	0.80
*l*	0.50	0.59	0.67	0.53	0.63	0.71	0.61	0.68	0.80
*p*_*excess*_	0.37	0.51	0.56	0.44	0.52	0.63	0.55	0.60	0.68
5%									
*l*_*KL*_	0.56	0.64	0.71	0.61	0.67	0.77	0.67	0.75	0.83
*l*	0.56	0.64	0.71	0.61	0.67	0.77	0.67	0.75	0.83
*p*_*excess*_	0.41	0.55	0.63	0.48	0.51	0.65	0.59	0.68	0.72

Note: The MAF of the causal variant is 0.01(*P*_*a*_ = 0.01). The sample size is 1500 (2 *N* = 1500)

## Discussion

In this report, we present a robust approach to identify rare variant of quantitative traits. The proposed approach adopts phenotype extreme selection design and KL-distance method. We use a two-stage strategy in our analysis where the first stage is association analysis and the second stage is fine mapping of QTL if the first stage is positive result. We propose a statistic T_KL_ for association analysis and a LD measure *l*_*KL*_ for fine mapping. Simulation studies present the performance of the proposed method. We found that the power of the T_KL_ increases with the stringency of the sample selection and the increasing of the number of causal variants. The T_KL_ here has higher power for association analysis than three existing statistics. Meanwhile, the impact of non-causal variants and the opposite effect variants on the T_KL_ is slight. The LD measure *l*_*KL*_ for fine mapping in the second stage has a good feature of not dependence on the frequencies of non-causal variants and just dependence on the frequencies of causal variants. These results show that our method can be used to detect rare variant associated with quantitative traits. At the same time, we found that the proposed method can be easily extended to case-control study by treating cases and controls as samples with upper extreme phenotype and lower extreme phenotype, respectively.

In rare variant association analysis, in order to achieve high statistical power of tests, usually a large sample with high sequencing costs is needed. Thus less costly sequencing design is preferred in rare variant association study. For quantitative traits, extreme phenotypes are generally considered to be more informative because of rare causal variants enriched among them. One can use a smaller sample size for extreme-phenotype sampling to achieve similar power as that for random sampling [23, 24]. Moreover, because extreme phenotypes of quantitative traits relative to human health are of primary clinical significance and thus data set can be obtained easily for subjects with extreme phenotypic values, using extreme phenotype samples in association analysis will make our study useful and practical. Here we use KL-distance to construct the statistics T_KL_ to measure the difference between two probability distributions of rare variants in two extreme populations. Based on the principle that the larger T_KL_ value is, the more dissimilar two probability distributions of rare variants, the statistics T_KL_ can be used as a test statistic to quantify the magnitude of association between the variants and the quantitative trait in the first stage of association analysis. We found that the statistic T_KL_ here for association analysis has higher power than three existing statistics of the burden test, the SKAT, and the SKAT-O. Moreover, whereas increasing the number of non-causal variants and the opposite effect variants result in decreasing severely the powers of the burden test, the SKAT, and the SKAT-O, non-causal variants and the opposite effect variants affect slightly on the T_KL_. The T_KL_ has relatively stable power with small change range under various parameters set.

In the second stage of fine mapping, *l*_*KL*_ is essentially a measure of LD between the variant and the QTL. Although LD between rare variant and QTL maybe weak [24], the maximum value across all rare variants can be usually found to identify the causal variant (QTL). The measure *l*_*KL*_ here has a good performance of just dependence on the frequency of the causal variant. In practice, not dependence on the frequency of the non-causal variant can eliminate“noise” and even bias introduced by varying frequencies of non-causal variants. In our early works, we proposed the LD measure *l* for mapping common variant of the QTL [27]. The performance of the measure *l* for mapping rare variant is unknown. We found from theory analysis that the two LD measures *l*_*KL*_ and *l* are parallel and have the same performance, that is, both of them can quantify LD between the variant and the QTL and do not depend on frequencies of non-causal variants. The difference between them is that the measure *l*_*KL*_ here is based on KL-distance and the measure *l* is based on entropy theory. Another LD measure for fine mapping is *p*_*excess*_ [28]. The *p*_*excess*_ is originally developed for fine mapping common variant of qualitative trait. We compare the performance of these three LD measures for fine mapping rare variant of quantitative traits using extreme samples. We found from theory analysis and simulation study that *l*_*KL*_ is superior to *p*_*excess*_.

It is noted that, in practice, we do not know how many causal variants there are in the region established through association analysis at first stage. Although we considered a region having only a single causal variant, our method works for the general case with a region consisting of multiple causal variants. In fact, when there is a region linked to a quantitative trait has multiple causal variants, we can detect all causal variants using following steps: (1) *l*_*KL*_ is used to mapping a causal variant with the maximum value of *l*_*KL*_; (2) T_KL_ is used to do association analysis for all variants except the causal variant detected in (1). If the association analysis result is positive, then return to (1). All causal variants will be found when the association analysis result is negative. It should be noted that we use the permutation procedure to assess the statistical significance of the statistic T_KL_ for association analysis. Permutation procedure may need more computing time to conduct simulation. But with the development of high-performance computing, computing time may not be a problem in our study. In addition, it can be seen that our method involves only rare variants. A phenotype may affected by common variants or both common variants and rare variants. So our further work will involve extensive field for common variants or both common variants and rare variants.

## Conclusions

The statistic T_KL_ is affected slightly by non-causal variants and the opposite effect variants. The power of the T_KL_ for association analysis of rare variants increases with the stringency of the sample selection for quantitative traits. Extreme phenotypes allow T_KL_ to achieve higher power than three commonly used methods. The LD measure *l*_*KL*_ for fine mapping is independent of the frequencies of non-causal variants and just dependent on the frequencies of causal variants.

## Methods

In this study, all datasets were publically available and no research requiring ethics approval was conducted.

We consider an interesting gene region with *k* biallelic variants and assume that each variant has a minor allele *m* with the MAF *P*_*m*_ and a normal allele *M* with the allele frequency *P*_*M*_ (*P*_*m*_ + *P*_*M*_ = 1). The variants are indexed by *i* (*i* = 1, ..., *k*). The index *i* may or may not correspond to the variant orders. Let *X*_*i*_ be minor allele count at *i*th variant carried by a subject. Assume that there is a quantitative trait Y: Y = *β*_0_ + *G* + *ε*, where $G=\underset{i=1}{\overset{k}{\Sigma}}{\beta}_i{X}_i$, *β*_0_ is the mean baseline value, and *ε* is residual due to random environmental effects. Without loss of generality, we assume *β*_0_ =0 and *ε*~*N*(0, *σ*^2^). To simplify our presentation, we use a measure with a superscript “U” to indicate a measure in the upper extreme population that has phenotypic values of the quantitative trait *Y* > *U* (*U* is an upper-threshold value, chosen from the continuous distribution of the study quantitative trait). We also use a measure with a superscript “L” to indicate a measure in the low extreme population that has phenotypic values of the quantitative trait *Y* < *L* (*L* is an low-threshold value, chosen from the continuous distribution of the study quantitative trait). Assume N^U^ and N^L^ subjects are sequenced with *k* variants in the upper extreme population and in the low extreme population, respectively, which are indexed by *j* (*j* = 1,..., N^U^/ N^L^). Denote ${X}_{ij}^{\mathrm{U}}$ and ${X}_{ij}^{\mathrm{L}}$ as the number of copies “*m”* for *j*th subject at *i*th variant in the upper extreme population and in the low extreme population, respectively. Then the frequencies of *P*_*m*_ and *P*_*M*_ at *i*th variant in the upper extreme population and in the low extreme population, denoted as ${p}_{mi}^{\mathrm{U}}$, ${p}_{Mi}^{\mathrm{U}}$, and ${p}_{mi}^{\mathrm{L}}$, ${p}_{Mi}^{\mathrm{L}}$, respectively, are estimated as follows:

$$ {p}_{mi}^{\mathrm{U}}=\frac{\underset{j=1}{\overset{N^{\mathrm{U}}}{\Sigma}}{X}_{ij}^{\mathrm{U}}}{2{N}^{\mathrm{U}}} $$, $$ {p}_{Mi}^{\mathrm{U}}=1-{p}_{mi}^{\mathrm{U}} $$, $$ {p}_{mi}^{\mathrm{L}}=\frac{\underset{j=1}{\overset{N^{\mathrm{L}}}{\Sigma}}{X}_{ij}^{\mathrm{L}}}{2{N}^{\mathrm{L}}} $$, and $$ {p}_{Mi}^{\mathrm{L}}=1-{p}_{mi}^{\mathrm{L}} $$.

### A statistic test for association analysis in the first stage

In the first stage, we propose a statistic test for association analysis. We define a k-dimensional random vector ${\tilde{p}}_m={\left({\tilde{p}}_{m1},\cdots, {\tilde{p}}_{mk}\right)}^T$ as the proportion of the minor allele *m* among all *k* variants, where ${\tilde{p}}_{mi}=\frac{\underset{j}{\Sigma}{X}_{ij}}{\underset{i=1}{\overset{k}{\Sigma}}\underset{j}{\Sigma}{X}_{ij}}$ and *X*_*ij*_ is the number of copies “*m”* for *j*th subject at *i*th variant. In the upper extreme population and in the low extreme population, the k-dimensional random vector of the proportion of the minor allele *m* are denoted as ${\tilde{p}}_m^{\mathrm{U}}={\left({\tilde{p}}_{m1}^{\mathrm{U}},\cdots, {\tilde{p}}_{mk}^{\mathrm{U}}\right)}^T$ and ${\tilde{p}}_m^{\mathrm{L}}={\left({\tilde{p}}_{m1}^{\mathrm{L}},\cdots, {\tilde{p}}_{mk}^{\mathrm{L}}\right)}^T$, respectively, where ${\tilde{p}}_{mi}^{\mathrm{U}}=\frac{\underset{j=1}{\overset{N^{\mathrm{U}}}{\Sigma}}{X}_{ij}^{\mathrm{U}}}{\underset{i=1}{\overset{k}{\Sigma}}\underset{j=1}{\overset{N^{\mathrm{U}}}{\Sigma}}{X}_{ij}^{\mathrm{U}}}$ and ${\tilde{p}}_{mi}^{\mathrm{L}}=\frac{\underset{j=1}{\overset{N^{\mathrm{L}}}{\Sigma}}{X}_{ij}^{\mathrm{L}}}{\underset{i=1}{\overset{k}{\Sigma}}\underset{j=1}{\overset{N^{\mathrm{L}}}{\Sigma}}{X}_{ij}^{\mathrm{L}}}$ (*i* = 1, 2, ⋯, *k*). We compare the two probability distributions ${\tilde{p}}_m^{\mathrm{U}}$ and ${\tilde{p}}_m^{\mathrm{L}}$ using the KL-distance which is defined as in Kullback & Leibler [26], here, we denote it the statistic T_KL_:

1 $$ {\mathrm{T}}_{KL}=H\left({\tilde{p}}_m^{\mathrm{U}},{\tilde{p}}_m^{\mathrm{L}}\right)=\frac{1}{2}\left(\underset{i=1}{\overset{k}{\Sigma}}{\tilde{p}}_{mi}^{\mathrm{U}}\cdot \log \frac{{\tilde{p}}_{mi}^{\mathrm{U}}}{{\tilde{p}}_{mi}^{\mathrm{L}}}+\underset{i=1}{\overset{k}{\Sigma}}{\tilde{p}}_{mi}^{\mathrm{L}}\cdot \log \frac{{\tilde{p}}_{mi}^{\mathrm{L}}}{{\tilde{p}}_{mi}^{\mathrm{U}}}\right) $$

It is easy to find the relationship between T_KL_ and the frequencies of *P*_*m*_ and *P*_*M*_ as follows:

2 $$ {\mathrm{T}}_{\mathrm{KL}}=\frac{1}{2}\left(\underset{i=1}{\overset{k}{\Sigma}}\frac{p_{mi}^{\mathrm{U}}}{\underset{i=1}{\overset{k}{\Sigma}}{p}_{mi}^{\mathrm{U}}}\cdot \log \left(\frac{p_{mi}^{\mathrm{U}}}{p_{mi}^{\mathrm{L}}}\cdot \frac{\underset{i=1}{\overset{k}{\Sigma}}{p}_{mi}^{\mathrm{L}}}{\underset{i=1}{\overset{k}{\Sigma}}{p}_{mi}^{\mathrm{U}}}\right)+\underset{i=1}{\overset{k}{\Sigma}}\frac{p_{mi}^{\mathrm{U}}}{\underset{i=1}{\overset{k}{\Sigma}}{p}_{mi}^{\mathrm{U}}}\cdot \log \left(\frac{p_{mi}^{\mathrm{L}}}{p_{mi}^{\mathrm{U}}}\cdot \frac{\underset{i=1}{\overset{k}{\Sigma}}{p}_{mi}^{\mathrm{U}}}{\underset{i=1}{\overset{k}{\Sigma}}{p}_{mi}^{\mathrm{L}}}\right)\right) $$

T_KL_ is the mean between two KL-distances where one is the KL-distance between ${\tilde{p}}_m^{\mathrm{U}}$ and ${\tilde{p}}_m^{\mathrm{L}}$ and the other is the KL-distance between ${\tilde{p}}_m^{\mathrm{L}}$ and ${\tilde{p}}_m^{\mathrm{U}}$. KL-distance provides a non-symmetric measure of how big of the difference between two probability distributions are. The KL-distance is always non-negative and equal to 0 only if two distributions are identical. It can be seen that T_KL_ is a non-negative and symmetric measure of the two probability distributions ${\tilde{p}}_m^{\mathrm{U}}$ and ${\tilde{p}}_m^{\mathrm{L}}$. So, T_KL_ can be used as a statistic to quantify the magnitude of association between the variants and the quantitative trait: a much larger T_KL_ value will be observed under the alternative hypothesis of association compared to that under the null hypothesis of no association.

### A KL-distance index for fine mapping of QTL in the second stage

Assume that a region linked to a quantitative trait has already been established through association analysis at first stage. In order to simplify our presentation, we assume that this region contains only a causal variant with a minor allele *a* (with frequency *p*_*a*_) and a normal allele *A* (with frequency *p*_*A*_ = 1 − *p*_*a*_), here, we call it the quantitative trait locus (QTL). We consider the quantitative trait Y = *G*_*Q*_ + *ε*, where *G*_*Q*_ is the genotypic value at the QTL and *ε*~*N*(0, *σ*^2^). We hope to fine map this region by calculating the linkage disequilibrium (LD) measure between the QTL and a variant. We still use KL-distance to construct this measure through comparing the probability distributions of allele *m* and *M* at a variant in the upper extreme population and in the low extreme population. Following the previous symbols, let *P*_*m*_ and *P*_*M*_ be the frequencies of allele *m* and *M* at a variant. From Eq. (1), we have

3 $$ H\left(\left\{{p}_m^{\mathrm{U}},{p}_M^{\mathrm{U}}\right\},\left\{{p}_m^{\mathrm{L}},{p}_M^{\mathrm{L}}\right\}\right)=\frac{1}{2}\left({p}_m^{\mathrm{U}}\cdot \log \frac{p_m^{\mathrm{U}}}{p_m^{\mathrm{L}}}+{p}_M^{\mathrm{U}}\cdot \log \frac{p_M^{\mathrm{U}}}{p_M^{\mathrm{L}}}+{p}_m^{\mathrm{L}}\cdot \log \frac{p_m^{\mathrm{L}}}{p_m^{\mathrm{U}}}+{p}_M^{\mathrm{L}}\cdot \log \frac{p_M^{\mathrm{L}}}{p_M^{\mathrm{U}}}\right) $$

From Appendix, $H\left(\left\{{p}_m^{\mathrm{U}},{p}_M^{\mathrm{U}}\right\},\left\{{p}_m^{\mathrm{L}},{p}_M^{\mathrm{L}}\right\}\right)$ can be asymptotically expressed as a function of LD (*δ*_*am*_) between the QTL and the variant:

4 $$ H\left(\left\{{p}_m^{\mathrm{U}},{p}_M^{\mathrm{U}}\right\},\left\{{p}_m^{\mathrm{L}},{p}_M^{\mathrm{L}}\right\}\right)\approx \frac{\delta_{ma}^2\cdot {\left({b}_U-{b}_L\right)}^2}{2{p}_m\cdot {p}_M} $$

Assume that there is an initial complete association between the variant allele *m* and the QTL allele *a*, at the 0th generation when the allele *a* is initially introduced into the study population. Let ${\delta}_{ma}^{(0)}$ be the initial complete LD between the allele *a* and *m* at the 0th generation, ${\delta}_{ma}^{(0)}={p}_M\cdot {p}_a$. After n generations, the LD between the allele *m* and *a* is ${\delta}_{ma}^{(n)}={\left(1-\theta \right)}^n{\delta}_{ma}^{(0)}={\left(1-\theta \right)}^n\cdot {p}_M\cdot {p}_a$ [29], where, *θ* is the recombination between the QTL and the variant. Then we have

5 $$ H\left(\left\{{p}_m^{\mathrm{U}},{p}_M^{\mathrm{U}}\right\},\left\{{p}_m^{\mathrm{L}},{p}_M^{\mathrm{L}}\right\}\right)\approx \frac{\delta_{am}^2\cdot {\left({b}_U-{b}_L\right)}^2}{2{p}_M\cdot {p}_m}=\frac{{\left(1-\theta \right)}^{2n}{p}_a^2\cdot {p}_M^2\cdot {\left({b}_U-{b}_L\right)}^2}{2{p}_M\cdot {p}_m} $$

Now we define a LD measure, here, we denote it as *l*_*KL*_, as follows:

6 $$ {l}_{KL}=\frac{p_m}{p_M}H\ \left(\left\{{p_m}^{\mathrm{U}},{p_M}^{\mathrm{U}}\right\},\left\{{p_m}^{\mathrm{L}},{p_M}^{\mathrm{L}}\right\}\right)\approx \frac{1}{2}{\left(1-\theta \right)}^{2n}{p}_a^2\cdot {b}^2 $$

where *b* = *b*_*U*_ − *b*_*L*_. It can be seen that *l*_*KL*_ is a decreasing function of the recombination *θ* and reaches its maximum at *θ* = 0. So we can use *l*_*KL*_ to find the variant closest to the QTL and thus fine map the QTL. Notice from Eq. (6) that *l*_*KL*_ is independent of the frequency of the variant, just only dependent on the frequency of the QTL.

### Simulation

#### Simulation for association analysis

To evaluate the performance of the test statistic T_KL_, we perform a series of simulation studies. We consider k = 100 variants with MAF values of causal variants determined by a uniform distribution *U* (0.001, 0.01) and MAF values of non-causal variants determined by a uniform distribution *U* (0.001, 0.05). The genotype data are simulated similar to those in Wang and Elston [30]. We first generate haplotypes for k variants based on a latent variable Z = (*Z*_1_, ⋯, *Z*_*k*_) from a multivariate normal distribution with covariance structure cov(*Z*_*i*_, *Z*_*j*_) = 0.4^∣*i* − *j*∣^ between any two latent components. Then we combine two haplotypes to obtain the genotype value for each individual *X*_*i*_ = (*X*_*i*1_, ⋯, *X*_*ik*_). A phenotype Y under the null hypothesis of no association is generated using the model Y = *ε* with *ε*~*N*(0, 1) (*β*_1_ = ⋯ = *β*_*k*_ = 0). Under the alternative hypothesis of association, we randomly chose s variants as causal variants while other k-*s* variants as non-causal variants having *β*_*j*_ = 0. Here, we let s = 10, 20, 50 in which 10, 20%, or 50% of rare variants were causal. For causal variants under the alternative hypothesis, we set *β*_*j*_ = c ⋅ log_10_(*p*_*mi*_) as used in Lee et al. [10], where c is 0.6, 0.3, and 0.2 for different values of s and different direction of the effects of causal variants. We consider 9 scenarios in the simulation study with the parameter values detailed in Table 3. We conduct 1000 simulations for each scenario. In each simulation, we select three extreme sample strategies, the low 20% and the up 20%, the low 10% and the up 10%, and, the low 5% and the up 5%, each of which consists of 2 N individuals including N individuals in an upper sample and N individuals in a lower sample. The statistical significance is assessed by a permutation procedure. We first calculate the value of the data-based statistic T_KL_ for each simulation. Then we permute the “upper sample” and “lower sample” labels with equal probability and recalculate the statistic T_KL_ for 1000 times. The estimated *P* value is then the proportion of permutation-based statistics that are larger than the data-based statistic in 1000 permutations for each simulation. For a given significance level *α*, the power/type I error rate is estimated as the proportion of rejecting the null hypothesis when *p*-value ≤ *α* in 1000 simulations. In order to compare the performance of the test statistic T_KL_ with the existing methods, we also obtain the power for the burden, SKAT, and SKAT-O tests using case-control design with the same samples as for the test statistic T_KL_.

**Table 3:** The parameter values for power study

Scenario	causal variants (s)	Effect size weights (c)	Positive direction: Negative direction
1	s = 10	c = 0.6	10:0
2	s = 20	c = 0.3	20:0
3	s = 50	c = 0.2	50:0
4	s = 10	c = 0.6	8:2
5	s = 20	c = 0.3	16:4
6	s = 50	c = 0.2	40:10
7	s = 10	c = 0.6	5:5
8	s = 20	c = 0.3	10:10
9	s = 50	c = 0.2	25:25

#### Simulation for fine mapping

To assess the performance of the LD measure *l*_*KL*_ in fine mapping rare causal variants of quantitative traits, we conduct a simulation study using the method similar to those described in our early work [27, 28]. We consider a genetic region that has 21 variants, where only a variant locating at the middle of variant 10 and variant 11 is causal variant (that is, the QTL). The MAF of the causal variant is set to be 0.01(*p*_*a*_ =0.01) and the MAFs for 20 other variants are uniformly determined with values ranging from 0.001 to 0.05. Other parameters in simulation include the ratio d/v (here, v and d are the genotypic values for individuals with genotypes *aa* and *Aa*, respectively), the thresholds *L* and *U*, the heritability (h^2^) of the causal variant, and the sample size (2 N) [31]. We let the ratio d/v be − 1, 0, and 1 which correspond to recessive, additive, and dominant models, respectively. Once the parameter values are chosen, a population with the effective size of 15,000 is simulated starting from the 0th generation, with an initial complete association between the minor allele *a* at the causal variant and *m* at other variants [P(*m***|***a*) = 1]. The population then evolved for 50 generations under random mating and genetic drift. A hundred populations are simulated for analyses.

## Data Availability

All data generated or analyzed during this study are included in this published article.

## Appendix

Firstly, we calculate ${p}_m^{\mathrm{U}}\cdot \log \frac{p_m^{\mathrm{U}}}{p_m^{\mathrm{L}}}$. Under the assumption of random mating and, thus, Hardy-Weinberg (HW) equilibrium holding in the population, we can get.

$$ {p}_m^{\mathrm{L}}= pr\left(m|Y<L\right)= pr\left(m,Y<L\right)/{\varphi}_L $$, where *φ*_*L*_ = *pr*(*Y* < *L*)

$$ {\displaystyle \begin{array}{l} pr\left(m,Y<L\right)= pr\left(m, aa,Y<L\right)+ pr\left(m, aA,s<T\right)+ pr\left(m, AA,Y<L\right)\\ {}={p}_{ma}\cdot {p}_a\cdot pr\left(Y<L| aa\right)+\left({p}_{ma}\cdot {p}_A+{p}_{mA}\cdot {p}_a\right)\cdot pr\left(Y<L| aA\right)+{p}_{mA}\cdot {p}_A\cdot pr\left(Y<L| AA\right)\\ {}={p}_{ma}\cdot {p}_a\cdot {\phi}_{11}+\left({p}_{ma}\cdot {p}_A+{p}_{mA}\cdot {p}_a\right)\cdot {\phi}_{12}+{p}_{mA}\cdot {p}_A\cdot {\phi}_{22}\\ {}={a}_1\cdot {p}_{ma}+{a}_2\cdot {p}_{mA}\end{array}} $$

where *ϕ*_11_ = *pr*(*Y* < *L*| *aa*), *ϕ*_12_ = *pr*(*Y* < *L*| *aA*), *ϕ*_22_ = *pr*(*Y* < *L*| *AA*), *a*_1_ = (*ϕ*_11_ ⋅ *p*_*a*_ + *ϕ*_12_ ⋅ *p*_*A*_)/*φ*_*L*_, *a*_2_ = (*ϕ*_22_ ⋅ *p*_*A*_ + *ϕ*_12_ ⋅ *p*_*a*_)/*φ*_*L*_.

Note that.

*p*_*ma*_ = *p*_*m*_ ⋅ *p*_*a*_ + *δ*_*ma*_,   *p*_*mA*_ = *p*_*m*_ ⋅ *p*_*A*_ + *δ*_*mA*_, *δ*_*ma*_ =  − *δ*_*mA*_ *and  a*_1_ ⋅ *p*_*a*_ + *a*_2_ ⋅ *p*_*A*_ = 1, here *δ*_*m*~_ is the measure of LD between variant allele *m* and the QTL allele *~* and is defined as *δ*_~*m*_ = *p*_~*m*_ − *p*_~_ ⋅ *p*_*m*_, where *p*_*m*~_ is the frequency of haplotype *m ~* .

Then,

$$ {\displaystyle \begin{array}{l}{p}_m^L={a}_1\cdot \left({p}_m\cdot {p}_a+{\delta}_{ma}\right)+{a}_2\cdot \left({p}_m\cdot {p}_A+{\delta}_{mA}\right)={p}_m+{\delta}_{ma}\left({a}_1-{a}_2\right)\\ {}={p}_m+{b}_L\cdot {\delta}_{ma}\end{array}} $$

Where $$ {b}_L={a}_1-{a}_2 $$

Similarly, we can get ${p}_M^L={p}_M+{b}_L\cdot {\delta}_{Ma}$, ${p}_m^U={p}_m+{b}_U\cdot {\delta}_{ma}$ and ${p}_M^U={p}_M+{b}_U\cdot {\delta}_{Ma}$, where *b*_*U*_ = *c*_1_ − *c*_2_, *c*_1_ = (*γ*_11_ ⋅ *p*_*a*_ + *γ*_12_ ⋅ *p*_*A*_)/*φ*_*U*_, *c*_2_ = (*γ*_22_ ⋅ *p*_*A*_ + *γ*_12_ ⋅ *p*_*a*_)/*φ*_*U*_, *φ*_*U*_ = *pr*(*Y* > *U*),

*γ*_11_ = *pr*(*Y* > *U*| *aa*), *γ*_12_ = *pr*(*Y* > *U*| *aA*), *γ*_22_ = *pr*(*Y* > *U*| *AA*), *c*_1_ = (*γ*_11_ ⋅ *p*_*a*_ + *γ*_12_ ⋅ *p*_*A*_)/*φ*_*U*_, *c*_2_ = (*γ*_22_ ⋅ *p*_*A*_ + *γ*_12_ ⋅ *p*_*a*_)/*φ*_*U*_.

Then

$$ {\displaystyle \begin{array}{l}{p}_m^{\mathrm{U}}\cdot \log \frac{p_m^{\mathrm{U}}}{p_m^{\mathrm{L}}}={p}_m^{\mathrm{U}}\cdot \log {p}_m^{\mathrm{U}}-{p}_m^{\mathrm{U}}\cdot \log {p}_m^{\mathrm{L}}=\left({p}_m+{b}_U\cdot {\delta}_{ma}\right)\cdot \log \frac{p_m\cdot \left(1+{b}_U\cdot {\delta}_{ma}/{p}_m\right)}{p_m\cdot \left(1+{b}_L\cdot {\delta}_{ma}/{p}_m\right)}\\ {}=\left({p}_m+{b}_U\cdot {\delta}_{ma}\right)\cdot \left[\log \left(1+{b}_U\cdot {\delta}_{ma}/{p}_m\right)-\log \left(1+{b}_L\cdot {\delta}_{ma}/{p}_m\right)\right]\\ {}\left[ by\ \log \left(1+x\right)\approx x-{x}^2/2\right]\\ {}\approx \left({p}_m+{b}_U\cdot {\delta}_{ma}\right)\cdot \left[\left(\frac{b_U\cdot {\delta}_{ma}}{p_m}-\frac{{b_U}^2\cdot {\delta_{ma}}^2}{2{p_m}^2}\right)-\left(\frac{b_L\cdot {\delta}_{ma}}{p_m}-\frac{{b_L}^2\cdot {\delta_{ma}}^2}{2{p_m}^2}\right)\right]\\ {}=\left({p}_m+{b}_U\cdot {\delta}_{ma}\right)\cdot \frac{\left({b}_U-{b}_L\right)\cdot {\delta}_{ma}}{p_m}\cdot \left(1-\frac{\left({b}_U+{b}_L\right)\cdot {\delta}_{ma}}{2{p}_m}\right)\end{array}} $$

Similar

$$ {p}_M^{\mathrm{U}}\cdot \log \frac{p_M^{\mathrm{U}}}{p_M^{\mathrm{L}}}\approx \left({p}_M+{b}_U\cdot {\delta}_{Ma}\right)\cdot \frac{\left({b}_U-{b}_L\right)\cdot {\delta}_{Ma}}{p_M}\cdot \left(1-\frac{\left({b}_U+{b}_L\right)\cdot {\delta}_{Ma}}{2{p}_M}\right) $$

$$ {p}_m^{\mathrm{L}}\cdot \log \frac{p_m^{\mathrm{L}}}{p_m^{\mathrm{U}}}\approx \left({p}_m+{b}_L\cdot {\delta}_{ma}\right)\cdot \frac{\left({b}_L-{b}_U\right)\cdot {\delta}_{ma}}{p_m}\cdot \left(1-\frac{\left({b}_L+{b}_U\right)\cdot {\delta}_{ma}}{2{p}_m}\right) $$

$$ {p}_M^{\mathrm{L}}\cdot \log \frac{p_M^{\mathrm{L}}}{p_M^{\mathrm{U}}}\approx \left({p}_M+{b}_L\cdot {\delta}_{Ma}\right)\cdot \frac{\left({b}_L-{b}_U\right)\cdot {\delta}_{Ma}}{p_M}\cdot \left(1-\frac{\left({b}_L+{b}_U\right)\cdot {\delta}_{Ma}}{2{p}_M}\right) $$

Then

$$ {\displaystyle \begin{array}{l}H\left(\left\{{p}_m^{\mathrm{U}},{p}_M^{\mathrm{U}}\right\},\left\{{p}_m^{\mathrm{L}},{p}_M^{\mathrm{L}}\right\}\right)=\frac{1}{2}\left({p}_m^{\mathrm{U}}\cdot \log \frac{p_m^{\mathrm{U}}}{p_m^{\mathrm{L}}}+{p}_M^{\mathrm{U}}\cdot \log \frac{p_M^{\mathrm{U}}}{p_M^{\mathrm{L}}}+{p}_m^{\mathrm{L}}\cdot \log \frac{p_m^{\mathrm{L}}}{p_m^{\mathrm{U}}}+{p}_M^{\mathrm{L}}\cdot \log \frac{p_M^{\mathrm{L}}}{p_M^{\mathrm{U}}}\right)\\ {}\approx \frac{1}{2}\left[\frac{{\left({b}_U-{b}_L\right)}^2\cdot {\delta_{ma}}^2}{p_m}+\frac{{\left({b}_U-{b}_L\right)}^2\cdot {\delta_{Ma}}^2}{p_M}\right]\\ {}=\frac{{\delta_{ma}}^2\cdot {\left({b}_U-{b}_L\right)}^2}{2{p}_m\cdot {p}_M}\end{array}} $$

Assume that there is an initial complete association between the variant allele *m* and the QTL allele *a*, at the 0th generation when the allele *a* is initially introduced into the study population. Let ${\delta}_{ma}^{(0)}$ be the initial complete LD between the allele *a* and *m* at the 0th generation, ${\delta}_{ma}^{(0)}={p}_M\cdot {p}_a$. After n generations, the LD between the allele *m* and *a* is ${\delta}_{ma}^{(n)}={\left(1-\theta \right)}^n{\delta}_{ma}^{(0)}={\left(1-\theta \right)}^n\cdot {p}_M\cdot {p}_a$ [29], where, *θ* is the recombination between the QTL and the variant. Then we have

$$ H\left(\left\{{p}_m^{\mathrm{U}},{p}_M^{\mathrm{U}}\right\},\left\{{p}_m^{\mathrm{L}},{p}_M^{\mathrm{L}}\right\}\right)\approx \frac{{\delta_{ma}}^2\cdot {\left({b}_U-{b}_L\right)}^2}{2{p}_m\cdot {p}_M}=\frac{{\left(1-\theta \right)}^{2n}{p}_a^2\cdot {p}_M^2\cdot {\left({b}_U-{b}_L\right)}^2}{2{p}_m\cdot {p}_M} $$

Then, we have

$$ {l}_{KL}=\frac{p_m}{p_M}H\ \left(\left\{{p_m}^{\mathrm{U}},{p_M}^{\mathrm{U}}\right\},\left\{{p_m}^{\mathrm{L}},{p_M}^{\mathrm{L}}\right\}\right)\kern0.5em \approx \frac{1}{2}\ {\left(\ 1-\theta \right)}^{2n}{p}_a^2\cdot {b}^2 $$

where *b* = *b*_*U*_ − *b*_*L*_.

## Abbreviations

LD: Linkage disequilibrium
RV: Rare variant
KL-distance: Kullback-Leibler distance
SKAT: Sequence kernel association test
SKAT-O: The optimal test that combines SKAT and the burden test
QTL: Quantitative trait locus
HW: Hardy-Weinberg
